# Long Non-Coding RNAs Associated with Heterochromatin Function in Immune Cells in Psychosis

**DOI:** 10.3390/ncrna4040043

**Published:** 2018-12-18

**Authors:** Niyati Sudhalkar, Cherise Rosen, Jennifer K. Melbourne, Mi Rae Park, Kayla A. Chase, Rajiv P. Sharma

**Affiliations:** 1 Department of Psychiatry. University of Illinois at Chicago, Chicago, IL 60612, USA; nsudha2@uic.edu (N.S.); ccrosen@uic.edu (C.R.); jennikm@uic.edu (J.K.M.); mpark81@uic.edu (M.R.P.); kchase1@uic.edu (K.A.C.); 2 Department of Mental Health, Jesse Brown VA Medical Center, Chicago, IL 60612, USA

**Keywords:** lncRNAs, heterochromatin, antipsychotic response, chronic inflammation, schizophrenia, psychosis

## Abstract

Psychosis is associated with chronic immune dysregulation. Many long non-coding RNAs (lncRNAs) display abnormal expression during activation of immune responses, and play a role in heterochromatic regulation of gene promoters. We have measured lncRNAs MEG3, PINT and GAS5, selected for their previously described association with heterochromatin. Peripheral blood mononuclear cells (PBMCs) were isolated from blood samples collected from 86 participants with a diagnosis of psychosis and 44 control participants. Expression was assessed in relation to diagnosis, illness acuity status, and treatment with antipsychotic medication. We observed diagnostic differences with MEG3, PINT and GAS5, and symptom acuity effect with MEG3 and GAS5. Medication effects were evident in those currently on treatment with antipsychotics when compared to drug-naïve participants. We observed that clinical diagnosis and symptom acuity predict selected lncRNA expression. Particular noteworthy is the differential expression of MEG3 in drug naïve participants compared to those treated with risperidone. Additionally, an in vitro cell model using M2^tol^ macrophages was used to test the effects of the antipsychotic drug risperidone on the expression of these lncRNAs using quantitative real-time PCR (qRT-PCR). Significant but differential effects of risperidone were observed in M2^tol^ macrophages indicating a clear ability of antipsychotic medications to modify lncRNA expression.

## 1. Introduction

Schizophrenia, and psychosis in general, is associated with chronic immune dysregulation due to mechanisms that remain elusive [1,2]. In parallel, many long non-coding RNAs (lncRNAs) display abnormal expression during activation of immune responses by as yet unclear mechanisms, and thus it is likely that the chronic immune activation noted in psychosis will modify the expression of lncRNAs [3]. Concomitantly, lncRNAs physically interact with various RNA binding proteins (RBPs), several transcription factors and structural proteins that help in their functional regulation, altering gene transcription. [4].

**Figure 1:**
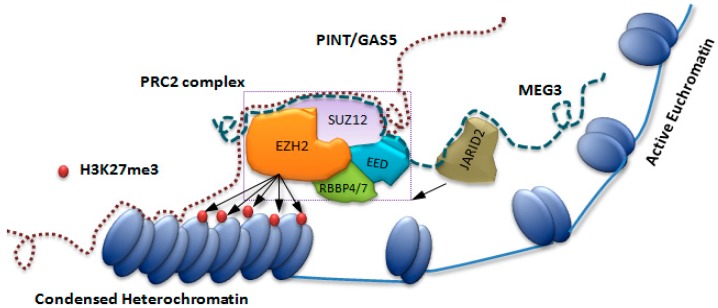
**Recruitment of PRC2 complex on the heterochromatin assembly.** Assembling the PRC2 components on sequence specific DNA mediated by lncRNAs (MEG3, PINT and GAS5). The PRC2 complex is shown with its core components (EZH2, SUZ12, EED and RBBP4/7) and this is responsible for di- and tri-methylation of nucleosomal histone proteins (H3K27me3). JARID2, a regulatory component of PRC2, interacts with the lncRNA MEG3 (blue dashed line) and helps the recruitment of the PRC2 to the chromatin. The lncRNAs (PINT or GAS5 denoted by the brown dotted line) cobble around the core component of PRC2 and help in its recruitment to the chromatin.

We have previously proposed that resistant epigenetic-heterochromatin assemblies are a putative underpinning for various long-term processes operative in individuals with psychosis, including chronic immunoreactivity [5]. LncRNAs are RNA transcripts that are longer than 200 nucleotides, with possibly more than 35,000 such transcripts expressed from across the genome. LncRNAs selectively mediate epigenetic modifications by recruiting chromatin remodeling complexes onto specific genomic loci. Polycomb Repressive Complex 2 (PRC2) is responsible in di- methylation and tri-methylation of lysine 27 in histone H3 (H3K27me2/3) which is a hallmark of facultative heterochromatin [6]. The core components of PRC2 (EZH2, EED, SUZ12, RBBP4/7) do not possess a DNA binding domain so the chromatin targeting may occur by chromatin-associated lncRNAs, as shown in Figure 1 [4,7]. As an illustration, JARID2, a regulatory component of the PRC2 complex, interacts with lncRNA MEG3 and this interaction facilitates the assembly of the entire complex on a genomic locus, as shown in Figure 1 [8,9].

Chronic inflammation can be modeled *in-vitro* with an extended application of the endotoxin lipopolysaccharide (LPS), resulting in the polarization of the M0 macrophage to the M2 tolerized phenotype, M2^tol^, by the assembly of heterochromatin along specific cytokine promoters. This heterochromatin structure is also quite possibly assembled by lncRNAs, as suggested by Figure 1 [3]. Our previous studies on the lncRNAs TMEVPG1 and NRON in participants with schizophrenia show that lncRNAs are responsive to inflammatory stimuli in a monocyte cell line, and have a relationship with atypical antipsychotics and pro-inflammatory cytokines [10].

The study of lncRNAs in psychiatric disorders is in its infancy and limited annotation of lncRNAs that are relevant to these disorders are available. Based on our resources, we were careful to select lncRNA molecules for study based on previously published annotated function. Selection criterion included structural links to heterochromatin assembly, and immune function relevant to samples of immune cells from participants with psychosis (schizophrenia and bipolar disorder).

To advance our understanding of heterochromatin function in psychosis, and the modification of expression of lncRNAs with antipsychotics, we selected three lncRNA molecules as per the criterion noted: MEG3, PINT and GAS5. Because lncRNAs can demonstrate strong tissue specific expression, we verified expression in purified primary CD14 monocytes using genome wide transcription RNA sequencing analysis [11].

Maternally expressed gene-3 (MEG3), also known as gene trap locus 2 (Gtl2), is a nuclear lncRNA, known to share common target genes with EZH2, the main subunit of PRC2 [12]. p53 induced transcript (PINT), directly interacts with PRC2, and is required for targeting specific genes for H3K27 tri-methylation and repression. The functional activity of PINT is highly dependent on PRC2 assembly and it is known to promote cell proliferation and survival by regulating the expression of genes of the TGF-β, MAPK and p53 pathways [13,14]. Similarly, growth arrest factor-5 (GAS5) is a nuclear lncRNA known to bind to EZH2, to inhibit M2 polarization in monocytes by suppressing IRF4 transcription. An altered methylation of H3K27 at the interferon regulatory factor 4 (IRF4) promoter is noted in M2 polarization. To the contrary, it is shown that interleukin 4 (IL-4) protein decreases GAS5 expression enhancing M2 monocyte/macrophage polarization [15].

This study aims to address the following questions: (1) What are the expression levels of lncRNAs MEG3, PINT and GAS5 in the context of diagnosis, clinical metrics and symptomatology in a sample of participants with psychosis (schizophrenia, bipolar disorder) and non-clinical controls? (2) What are the effects of antipsychotics on the expression of MEG3, PINT and GAS5 in human monocyte cell lines? (3) Can the findings from clinical samples be modeled in *in-vitro* cell-line experiments? 

## 2. Results

### 2.1. Demographics

Table 1 summarizes the sample characteristics of the total of 130 participants in this study. Of these, 86 participants were diagnosed with psychosis (schizophrenia: *n* = 63; bipolar disorder: *n* = 23). As shown in Table 1, there was a higher prevalence of smoking in the psychosis group, but no significant effect of smoking was found on the expression of any of the three lncRNAs. Table 2 details the Positive and Negative Syndrome Scale (PANSS five factor subscale scores) to assess illness acuity status. The correlation strength between the lncRNA (MEG3, PINT and GAS5) expression and illness severity as measured by the PANSS five factor is shown in Figures S1 and S2.

### 2.2. Diagnostic Differences

**Figure 2:**
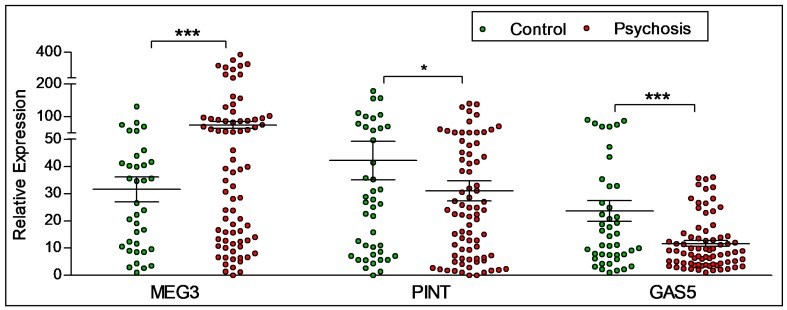
**Diagnostic difference in the expression of lncRNAs.** A difference in the expression of lncRNAs MEG3, PINT and GAS-5 was seen between control participants and participants with psychosis. Participants with psychosis had higher levels of MEG3, but lower levels of PINT and GAS5. Statistical significance is determined by *t*-test (* *p* < 0.05; *** *p* < 0.001). The figures represent Mean ± SEM for the relative expression of lncRNAs. SEM: Standard Error of Mean.

As shown in Figure 2, statistically significant diagnostic differences were observed in the expression levels of lncRNAs MEG3, PINT and GAS5. The expression of MEG3 was found to be higher in participants with psychosis (*n* = 86) when compared to control participants (*n* = 44). The expression levels of PINT and GAS5 decreased in participants with psychosis compared to control participants.

**Figure 3:**
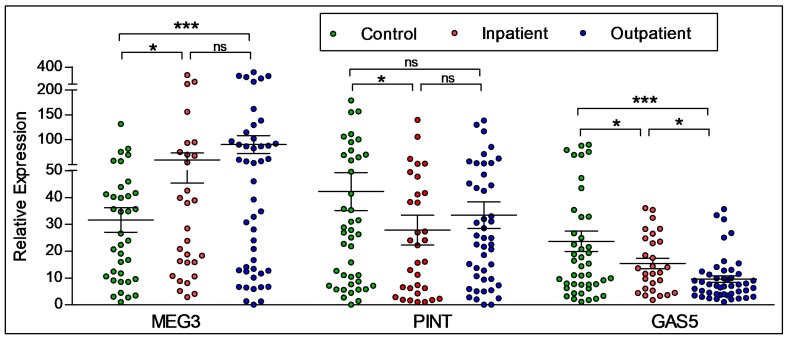
**LncRNA expression by illness acuity status.** Entire participant sample was used to determine the expression of the lncRNAs relative to illness acuity status. There were significant differences found in regard to inpatient or outpatient status. Statistical significance is determined using ANOVA (* *p* < 0.05; *** *p* < 0.001; ns, not significant). The figures represent Mean ± SEM for the relative expression of lncRNAs. SEM: Standard Error of Mean.

We next analyzed the effects of illness acuity in participants with psychosis (schizophrenia and bipolar disorder) in relation to their inpatient (*n* = 33) or outpatient (*n* = 53) status, and compared these groups to controls (*n* = 44) using a one-way ANOVA. As shown in Figure 3, significantly increased expression of the lncRNA MEG3 was seen in outpatients when compared to controls, as determined by Tukey’s post hoc tests. PINT expression was significantly lower in inpatients when compared to controls only, with no differences between any other condition. The expression of GAS5 was found to be significantly higher in controls when compared to both inpatients and outpatients, with higher expression found in inpatients compared to outpatients.

We also analyzed the ability of lncRNA (MEG3, PINT and GAS5) expression to predict clinical diagnosis and symptom acuity. A cluster dendrogram and heat map analyzed using hierarchical cluster analysis was generated based on the expression levels of all three lncRNAs combined. The results showed that, although not significant, there was a difference in the enrichment of clusters by diagnosis and illness acuity (Figure S3A,B). The receiver operating characteristic curve (ROC) validates the strength of the individual lncRNA for predicting clinical diagnosis and symptom acuity (Figure S4). We further analyzed the predictive power of individual lncRNA by conducting a discriminant analysis. Together we found that, of the three lncRNAs, MEG3 has a strong predictive power for diagnosis of psychosis. GAS5 is predictive of the control group characteristics and, we found that PINT has a predictive power (although not statistically significant) for the diagnosis of psychosis (Figure S4).

### 2.3. Co-Expression of lncRNAs

LncRNA expression was analyzed to examine the correlation with one another across the entire participant sample. LncRNA PINT was found to be significantly correlated with lncRNAs MEG3 and GAS5. On the other hand, there was no statistically significant correlation observed between the lncRNAs MEG3 and GAS5 (Table 3).

### 2.4. Differences in the Expression of lncRNAs Based on Antipsychotic Treatment Status in the Clinical Sample

**Figure 4:**
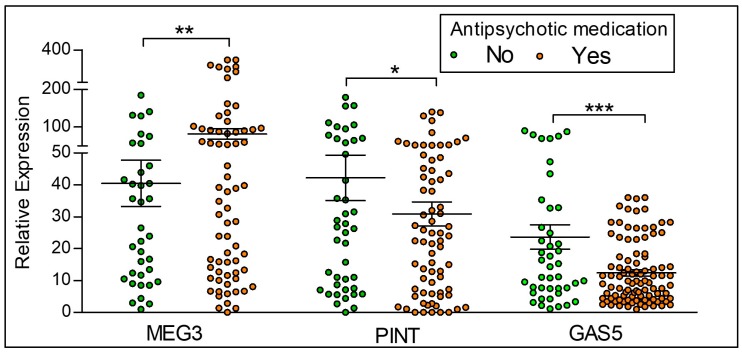
**LncRNA expression between controls and participants on antipsychotic medication**. Yes = Participants with the diagnosis of psychosis and currently on antipsychotic medication, No = Control participants who are not on antipsychotic medication. MEG3 is significantly higher, and PINT and GAS5 lower in those taking antipsychotic medication when compared to those not currently on antipsychotics. Statistical significance is determined by *t*-test (* *p* < 0.05; ** *p* < 0.01; *** *p* < 0.001; ns, not significant). The figures represent Mean ± SEM for the relative expression of lncRNAs. SEM: Standard Error of Mean.

Relative expression of the lncRNAs MEG3, PINT and GAS5 in participants with psychosis who are on *any* antipsychotic medication as compared to the control participants who are not on any antipsychotic treatment is shown in Figure 4. Of the 86 participants with a diagnosis of psychosis (schizophrenia and bipolar disorder), there are a total of 77 participants on an antipsychotic medication. None of the 44 controls received antipsychotic medication, as per our inclusion and exclusion criteria. We observe that antipsychotic medication is associated with significantly increased expression of MEG3, with significant decreases in PINT and GAS5 expression.

**Figure 5:**
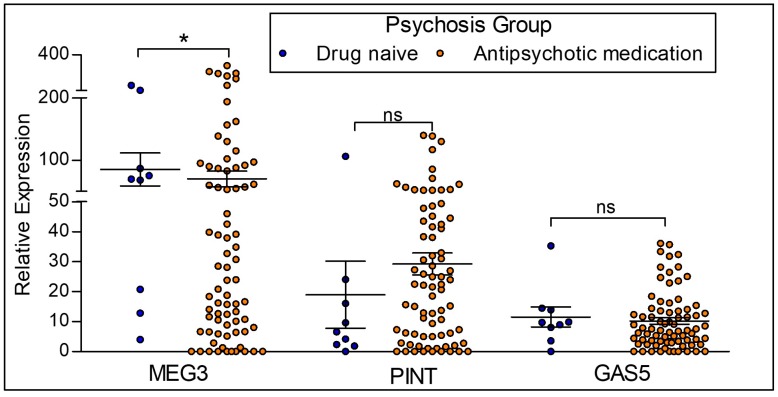
**LncRNA expression between drug naïve psychotic participants and participants on antipsychotic medication.** Antipsychotic medication = Participants with the diagnosis of psychosis and on antipsychotic medication. Drug naïve = Participants with a diagnosis of psychosis who are not on antipsychotic medication. MEG3 expression was significantly downregulated in participants currently on antipsychotic medication when compared to drug naïve psychotic participants. There were no differences in PINT and GAS5 expression levels between groups. Statistical significance is determined by one-way ANOVA (* *p* < 0.05; ns, not significant). The figures represent Mean ± SEM for the relative expression of lncRNAs. SEM: Standard Error of Mean.

To distinguish the effects of diagnosis from antipsychotic treatment, we examined drug-naïve participants with psychosis (*n* = 9) compared to participants with psychosis on antipsychotics (*n* = 77) (Figure 5). The expression of MEG3 is significantly reduced in participants with psychosis on antipsychotics when compared to the drug naïve participants with psychosis. There is no significant difference in the expression levels of lncRNAs PINT and GAS5.

**Figure 6:**
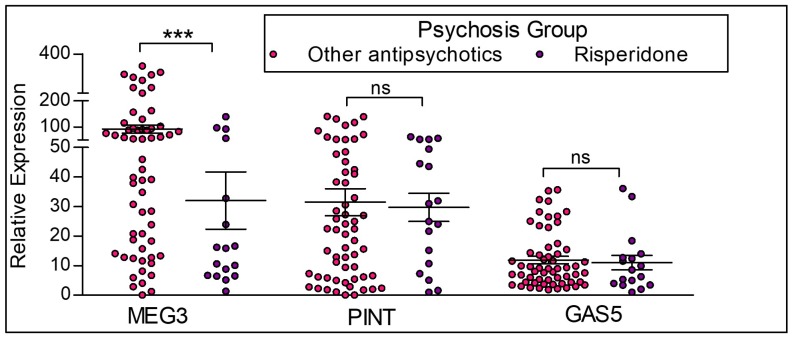
**LncRNA expression between participants with risperidone treatment and participants on other antipsychotic medications.** Risperidone = participants with the diagnosis of psychosis and on risperidone treatment alone. Other antipsychotics = participants with the diagnosis of psychosis who are not on risperidone medication, but on other antipsychotic medication. This group does not include drug naïve participants. Statistical significance is determined by one-way ANOVA and Tukey’s post hoc test (*** *p* < 0.001; ns, not significant). The figures represent Mean ± SEM for the relative expression of lncRNAs. SEM: Standard Error of Mean.

To examine the role of antipsychotic medication on lncRNA expression in the participants with psychosis, we assessed gene transcription changes in response to risperidone treatment (Figure 6). We compared the expression of lncRNAs between the participants currently on risperidone treatment alone (*n* = 19) versus participants currently on other antipsychotic treatment (*n* = 58; excluding the participants who were drug naïve). MEG3 has a significantly lower expression in participants with risperidone treatment compared to the participants not receiving risperidone treatment. In contrast, there were no differences in GAS5 and PINT expression levels between the groups.

**Figure 7:**
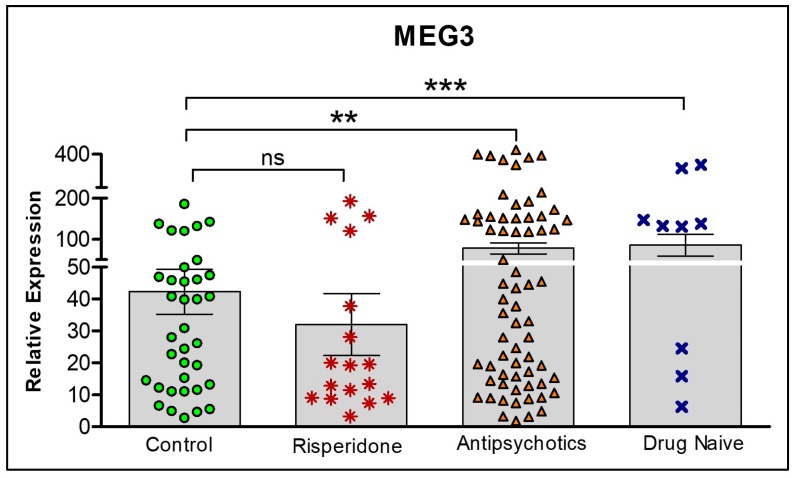
**Comparison of lncRNA MEG3 expression levels from four treatment conditions**: controls, risperidone only, currently on any other antipsychotic, and drug naïve. Statistical significance is determined by *t*-test (** *p* < 0.01; *** *p* < 0.001; ns, not significant). The figures represent Mean ± SEM for the relative expression of lncRNAs. SEM: Standard Error of Mean.

We further assessed the role of risperidone on MEG3 expression. Figure 7 shows differences in expression levels between all groups: Controls, risperidone only, other antipsychotics and drug naïve. MEG3 is highest in the drug naïve group, as determined by one-way ANOVA and Tukey’s post hoc tests. Interestingly, there are no statistical differences between controls and risperidone treated participants.

### 2.5. Effect of Antipsychotics on the expression of lncRNAs in the M0 and M2 macrophages

**Figure 8:**
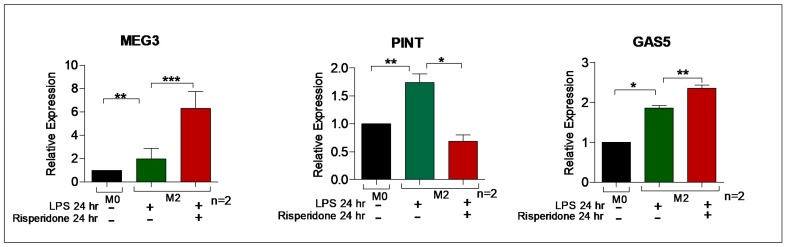
Antipsychotic response on induced M2^tol^ macrophages. MEG3 was significantly upregulated in response to LPS treatment, an effect compounded by Risperidone co-treatment, a pattern also seen with GAS-5. PINT was upregulated in response to LPS, but this effect was ablated with Risperidone treatment. The experiment was conducted in duplicate (*n* = 2), and M2^tol^ levels are compared to M0 set arbitrarily as the baseline. * *p* < 0.05; ** *p* < 0.01; *** *p* < 0.001; ns, not significant, as determined by ANOVA (*n* = 2).

Chronic inflammation can be modeled in vitro with an extended application of the LPS, resulting in endotoxin tolerance, or M2^tol^ characterized by the assembly of heterochromatin along specific cytokine promoters. The M2^tol^ macrophages were obtained by treating THP-1 derived M0 macrophages with LPS alone for 24 h, resulting in transcriptionally resistant heterochromatin formation to further immune stimulus. Risperidone has varying, but significant, effects in this chronic immune M2^tol^ paradigm, suppressing the expression of PINT, while increasing the expression of MEG3 and GAS5 (Figure 8), perhaps providing insight into pharmacological effects that are operative in the natural setting of clinical monocytes/macrophages.

## 3. Discussion

We observe both a diagnostic effect (for MEG3, PINT, GAS5) and a symptom acuity effect (for MEG3, GAS5) in differences of lncRNA expression levels between inpatients and outpatients versus controls. For the hierarchical clustering approach, as predicted, the cluster dendrogram was not able to group the samples as per diagnosis or symptom acuity when testing with all three lncRNAs together. This may suggest that these three lncRNAs are not coordinately operative along a particular signaling pathway involved in psychosis. Another explanation could be the mixture of cell phenotypes in a peripheral blood mononuclear cells (PBMC) sample, especially noting the tissue specific nature of lncRNA expression. It is nonetheless suggestive that the epigenetic effects in the chronic inflammatory pathway can be driven by each lncRNA individually, and not due to their combination.

Medication effects were demonstrated by the differences in gene expression between drug-naïve patients and those currently receiving antipsychotics, and this appears to be a normalizing effect for MEG3. It is evident in the elevated values for the ‘drug naïve participants’ compared to their antipsychotic treated counterparts. These effects vary according to the specific molecule, but are supported in an in vitro model demonstrating a clear pharmacological effect for risperidone, and allowing for the distinction between diagnosis and pharmacological treatment. Specifically, our results indicate that MEG3, a lncRNA that binds to a protein domain within JARID2 of the PRC2 repressome complex [9], is elevated in a subgroup of participants with psychosis. In myeloid cells, such as macrophages, MEG3 suppresses the activation of the PI3K/AKT pathway [16]. In humans, the proximal promoter has a highly conserved cAMP response element (CRE), which is activated by elevated cAMP intracellular levels, making it accessible to downstream G-protein signaling emanating from antipsychotic action [17]. MEG3 also has a Rel/NF-kB binding site ~300 bp upstream of the TSS suggesting regulation by incoming immune signals [17].

In contrast we find reduced levels of GAS5 expression in psychosis. GAS5 is a lncRNA with minimal sequence conservation of its exons, but with unambiguous biological function, partly dependent on highly conserved small nucleolar RNAs (snoRNAs) that are harbored within its introns. While all these molecules are capable of recruiting H3K4me4 histone methyltransferases and the H3K27me3 demethylase UTX (KDM6A), GAS5 can additionally act as decoy nucleotide binding site for the glucocorticoid receptor, implicating it in the etiology of psychiatric illnesses via this glucocorticoid mechanism [18]. These actions modify chromatin by inducing active chromatin through increased H3K4me3 and reduced H3K27me3 [19]. 

We found a marginal diagnostic difference in the expression of PINT, a lncRNA that directly interacts with the PRC2 heterochromatin complex (EZH2 component) [13]. The effects of antipsychotics on transcription level changes can be understood in several ways. An immediate effect is the ability of antipsychotics to accumulate intracellular levels of cAMP, and as noted above, MEG3 has a CRE element in its proximal promoter thereby explaining the relatively acute change seen in the M2^tol^ phenotype [20]. Over a longer period, antipsychotics, most clearly observed here with risperidone, reduces gene expression, possibly by an auto-regulatory obstruction of its CRE binding site. MEG3 regulates and enhances the specificity of binding of the transcription factor p53, and p53 in turn is a known regulator and activator of PINT expression [13]; coincidentally, this relationship is a possible explanation of the significant correlation between MEG3 and PINT expression seen in Table 3.

Differentiated THP-1 derived M2^tol^ allows us to examine antipsychotic effects in cell-types that are rarely accessible in living subjects. Tissue macrophages patrol at the boundary of the brain parenchyma (perivascular, meningeal and choroid plexus macrophages), communicate with parenchymal microglia, and influence GABA neuronal function [21,22]. These macrophages can consequently transmit signals across the boundary between peripheral and central nervous systems, and lncRNAs promise to be a major regulator of these cells’ genomic activities. In cells that have acquired heterochromatin (M2^tol^), the effects of risperidone for MEG3 and GAS5 raise an intriguing question as to whether chronic inflammation (modeled here with long-term LPS treatment) is associated with increases in lncRNAs in tissue macrophages.

Our results suggest that these lncRNAs (particularly, MEG3) are identified as plausible markers to study the epigenetic mechanisms involved in psychosis. As the study on lncRNAs in psychosis is at an early stage, it is likely that many other and possibly better candidates will be available in the future for analysis. Our studies indicate that specific well annotated lncRNAs have a potential to be considered for testing pharmacological effects and predicting clinical diagnosis and/ or symptom acuity in psychiatry research.

There are several challenges associated with the study of lncRNAs that we have attempted to address in this study. Sequence conservation does not always translate to functional conservation, which limits their experimentation in non-human tissues. LncRNAs are often species specific, with the level of conservation less when compared to protein coding genes, making our results measured in human tissues particularly relevant [23]. Additionally, the vast majority of lncRNAs have not been assigned a function, making the clinical applicability of this study difficult, but the study of lncRNAs in chronic psychiatric disorders is in its infancy, making the findings of this manuscript highly novel.

## 4. Materials and Methods

### 4.1. Participant Information and Clinical Measures

The study was approved by the institutional review board (IRB2012-0113, March 27, 2012), and signed consent obtained prior to study procedures. All procedures followed were in accordance with the ethical standards of the responsible committee on human experimentation (institutional and national) and with the Helsinki Declaration of 1975, as revised in 2000, 2014. Inclusion criteria included persons between the ages of 21 and 60 who met DSM-IV diagnostic criteria for schizophrenia, bipolar disorder with current psychotic symptoms, or controls with no history of a psychiatric disorder. Exclusion criteria included treatment with Valproic acid, Carbamazepine, or Clozapine in the previous 30 days, current substance dependence, seizure disorders, and neurological conditions. Consensus diagnoses were determined by RPS and CR (each with more than 20 years of clinical experience) using the Structured Clinical Interview for DSM-IVTR and available collateral information [24]. The Positive and Negative Syndrome Scale (PANSS) was used as the primary clinical measure.

### 4.2. Sample Collection, Processing and Quantitative real-time PCR (qRT-PCR)

Collection of blood samples, PBMC isolation, and RNA extraction were carried out according to previously described protocols [25]. RNA extracts were treated with DNase (Ambion®, California, USA) to remove any possible genomic DNA contaminants, and reverse transcribed using the High Capacity Archive Kit (Applied Biosystems, CA, USA). Maxima SYBR Green/ROX qPCR Master Mix (#K0222) was used for detection of PCR product and mixtures were run on a Thermo Scientific™ PikoReal System (UK). The samples were run in triplicates, and the relative quantification values were calculated using the Δ-Δ ct method relative to the geometric mean of the housekeeping genes GAPDH and ACTB [26]. Primers sequences are shown in Table 4. 

### 4.3. Primary Clinical Measures

Psychopathology was assessed using the positive and negative syndrome scale (PANSS) [27]. PANSS items were scored along a continuum of severity between 1 (asymptomatic) to 7 (extreme symptom severity). The coefficient alpha for inter-rater reliability was between 0.83 and 0.87. Analysis was conducted via data reduction strategies guided by prior empirical studies of symptom domains assessed by the PANSS. Scores were calculated for five-factors assessing Positive symptoms (delusions, grandiosity, suspiciousness/persecution, unusual thought content), Negative symptoms (blunted affect, emotional withdrawal, poor rapport, passive/apathetic social withdrawal, lack of spontaneity and flow of conversation, and active social avoidance), Cognitive Disorganization (conceptual disorganization, difficulty in abstract thinking, mannerisms and posturing, disorientation, and poor attention), Excitement (excitement, hostility, tension, and poor impulse control), and Depression (somatic concern, anxiety, guilt feelings, depression, and preoccupation). Items were grouped in this way based on previous factor analytic findings [28,29].

### 4.4. THP1-Derived M2^tol^ Polarized Macrophages and Risperidone Treatment

THP-1 cells (ATCC TIB-202), a human monocyte cell line, were maintained in culture at 37 °C and 5% CO2 with RPMI 1640 medium supplemented with 10% FBS, 2 mM Glutamine, and 50 u/mL each of penicillin and streptomycin. 6-well plates (1 × 10^6^ cells/well) were treated with 15 nM Phorbol 12-Myristate 13-Acetate (PMA; Sigma, Missouri, USA) for 24 h to induce differentiation into M0 macrophages. M0 macrophages were allowed to rest for 24 h prior to polarization [30]. We focus here on the M2 tolerized (M2^tol^) phenotype induced by high levels of the NF-κB activating stimulus lipopolysaccharide (LPS) [31,32]. For the M2^tol^ macrophage phenotype, the M0 cells were treated with 100 ng/uL LPS for 24 h to simulate the effects of chronic immune stimulation as might be seen in clinical patients. The effects of the antipsychotic drug risperidone on these macrophage phenotypes were tested by treating M2^tol^ macrophage cells with 10 µM risperidone or vehicle. RNA extraction and RT-qPCR (using biological duplicates) were carried out as previously described.

### 4.5. Statistical Analyses

**Figure 9:**
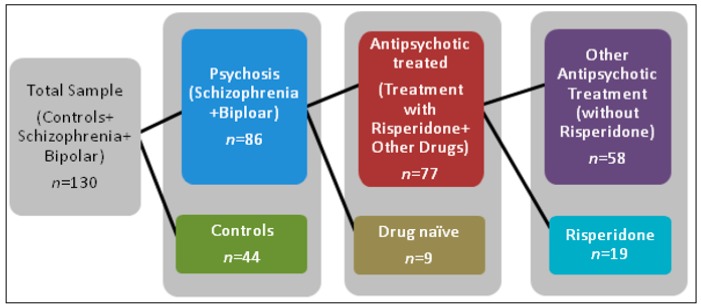
**Sample selection for analysis of lncRNA expression**. This diagram shows the total sample of study participants. First the total population was grouped by diagnosis. Then the psychosis group was further split by participants who received antipsychotic treatment and those participants who were drug naïve. The antipsychotic treated group was further sorted into those who received risperidone alone and participants who received all other antipsychotics except risperidone.

SPSS (version 24.0 for Windows) was used for statistical analyses. Group comparisons were analyzed using Chi Square, independent sample *t*-tests, and one-way ANOVAs with Tukey’s post-hoc tests to identify significant pair-wise group differences. Spearman correlations were used to determine associations between variables. Figure 9 shows the sample selection in terms of sample size for the analysis of lncRNA expression and antipsychotic response in the clinical setting. A multivariate linear discriminant analysis (using Mote–Carlo method) and a hierarchical cluster dendrogram (for complete-linkage between groups using Ward’s method at α = 0.95) with heat map was generated using STATA/SE 15.

## Figures and Tables

**Table 1:** Participant demographics for the control group and all participants with psychosis.

		Control		Psychosis		Group Differences
**Total (*n*)**		44 (33.8%)		86 (66.2%)		
**Sex**	Female (*n*)	25 (41.0%)		36 (59.0%)		χ^2^(3) = 2.615, *p* = 0.106
	Male (*n*)	19 (27.5%)		50 (72.5%)		
**Race**	Caucasian, non-Hispanic (*n*)	12 (54.5%)		10 (45.5%)		χ^2^(3) = 22.494, *p* < 0.001
	Black, non-Hispanic (*n*)	18 (22.5%)		62 (77.5%)		
	Asian or other Pacific Islander (*n*)	10 (83.3%)		2 (16.7%)		
	Hispanic (*n*)	4 (25.0%)		12 (75.0%)		
**Antipsychotic Use**	Yes (*n*)	0		77		
	No (*n*)	44		9		
Other medication *	Yes (*n*)	4		9		
	No (*n*)	40		77		
**Smoking**	Yes(*n*)	7		39		χ^2^(3) = 11.034, *p* = 0.001
	No (*n*)	37		47		
**Age (Mean ± SD)**		38.77 ± 12.82		40.21 ± 12.98		*t*(128) = −0.600, *p* = 0.55
**BMI (Mean ± SD)**		28.93 ± 8.1		30.8 ± 8.15		*t*(128) = 1.241, *p* = 0.217
		**Mean**	**SD**	**Mean**	**SD**	
**Age at onset of illness**		~	~	21.32	7.88	
**Duration of untreated psychosis**		~	~	2.97	5.85	
**Duration of illness**		~	~	19.33	14.35	

* Other medications indicate that the participant is concurrently taking a medication other than an antipsychotic, including hypertension, diabetes, etc. SD = Standard deviation. Age, age at onset of illness, duration of untreated psychosis and duration of illness are all reported in years.

**Table 2:** Participant clinical characteristics using PANSS five factor scores.

*N* = 130	Control (*n* = 44)		Psychosis (*n* = 86)				Group Differences	
			Inpatient(*n* = 33)		Outpatient(*n* = 53)			
PANSS Five-Factor Scores	Mean ± SD	Range	Mean ± SD	Range	Mean ± SD	Range	Inpatient and Outpatient ^†^	Control, Inpatient and Outpatient ^‡^
**Positive**	4.11 ± 0.49	4–7	18.27 ± 2.39	13–23	13.49 ± 4.40	5–21	*t*(84) = 5.73 ***	F(2,127) = 218.20 ***
**Negative**	6.55 ± 7.56	6–15	20.76 ± 7.56	6–36	15.60 ± 5.91	6–27	*t*(84) = 3.58 ***	F(2,127) = 68.56 ***
**Cognitive**	5.48 ± 1.07	5–10	17.34 ± 4.09	8–25	12.00 ± 4.01	5–21	*t*(84) = 5.95 ***	F(2,127) = 121.25 ***
**Excitement**	5.66 ± 1.98	4–13	10.91 ± 3.41	6–28	9.34 ± 2.78	4–16	*t*(84) = 2.33 *	F(2,127) = 39.35 ***
**Depression**	7.55 ± 2.71	5–14	15.69 ± 2.80	10–23	13.83 ± 3.33	6–22	*t*(84) = 2.68 **	F(2,127) = 83.173 ***

The table shows the illness acuity status for the control and psychosis groups. Five factor sub-scales of PANSS (Positive, Negative, Cognitive, Excitement and Depression) are shown for control participants and the participants with psychosis (categorized as Inpatient and Outpatient). SD = Standard deviation. ^†^ Two-tailed Independent sample *t*-tests for comparing group differences within psychosis groups-Inpatients and Outpatients ^‡^ ANOVA for comparing group differences between three groups-Controls, Inpatients and Outpatients. * *p* < 0.05; ** *p* < 0.01 *** *p* < 0.001.

**Table 3:** Correlation between lncRNAs.

	PINT	GAS5
**MEG3**	* r = 0.237 *p* = **0.015**	* r = 0.080 *p* = 0.422
**PINT**		* r = 0.222 *p* = **0.016**

* Spearman correlations.

**Table 4:** LncRNA Primer Sequences.

*GAPDH*	5′-CGAGATCCCTCCAAAATCAA-3′	5′-TTCACACCCATGACGAACAT-3′
*ACTB*	5′s-TGAAGGTAGTTTCGTGGATGC-3′	5′-TCCCTGGAGAAGAGCTACGA-3′
*MEG3*	5′-GCGGAGAGCAGAGAGGGA-3′	5′-AGGAGAGACCCGGGTGAG-3′
*PINT*	5′-CCATCTGGAGTTTCTCTGCCT-3′	5′-GGTAAGACTCTGTCTTCAGCTGTTA-3′
*GAS-5*	5′-TCCTGTGAGGTATGGTGCTGG-3′	5-AACTTGCCTGGACCAGCTTA-3′

## Supplementary Materials

The following are available online at http://www.mdpi.com/2311-553X/4/4/43/s1, Figure S1: Correlation between symptom severity and lncRNA expression. Figure S2: Correlational strength between lncRNA expression and disease severity (PANSS five factor sub-scale). Figure S3: Dendrograms and heat map of hierarchical clustering analysis (complete-linkage) by the expression of lncRNAs MEG-3, PINT and GAS-5. Figure S4: Receiver Operating Characteristic Curve (ROC) for predicting clinical diagnosis with lncRNAs (MEG-3, PINT and GAS-5) expression.
