# Supplementary material for: Long Non-Coding RNAs Associated with Heterochromatin Function in Immune Cells in Psychosis
Source: Noncoding RNA. 2018 Dec 18;4(4):43. doi: 10.3390/ncrna4040043 (PMC6316406; doi:10.3390/ncrna4040043)
Supplement: Supplementary file 1 [file ncrna-04-00043-s001.pdf]

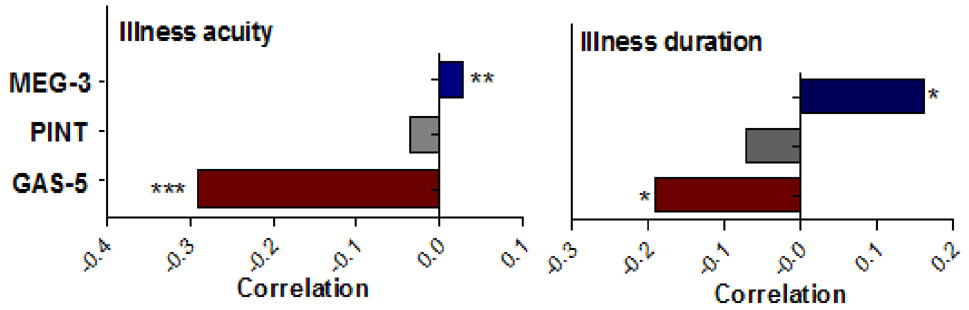

Figure S1. Correlation between symptom severity and lncRNA expression; *r=Spearman correlations;* *$p<0.05$; **$p\leq0.02$; ***$p\leq0.001$. *†Chronic conditions and first episode ‡Inpatients and outpatients.*

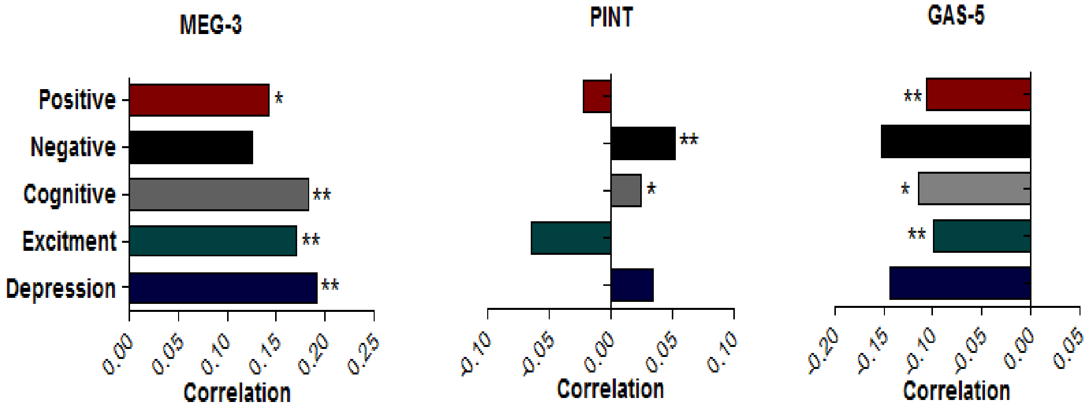

Figure S2. Correlational strength between lncRNA expression and disease severity (PANSS five factor sub-scale) *Spearman correlations* *$p<0.05$; **$p<0.02$.

A

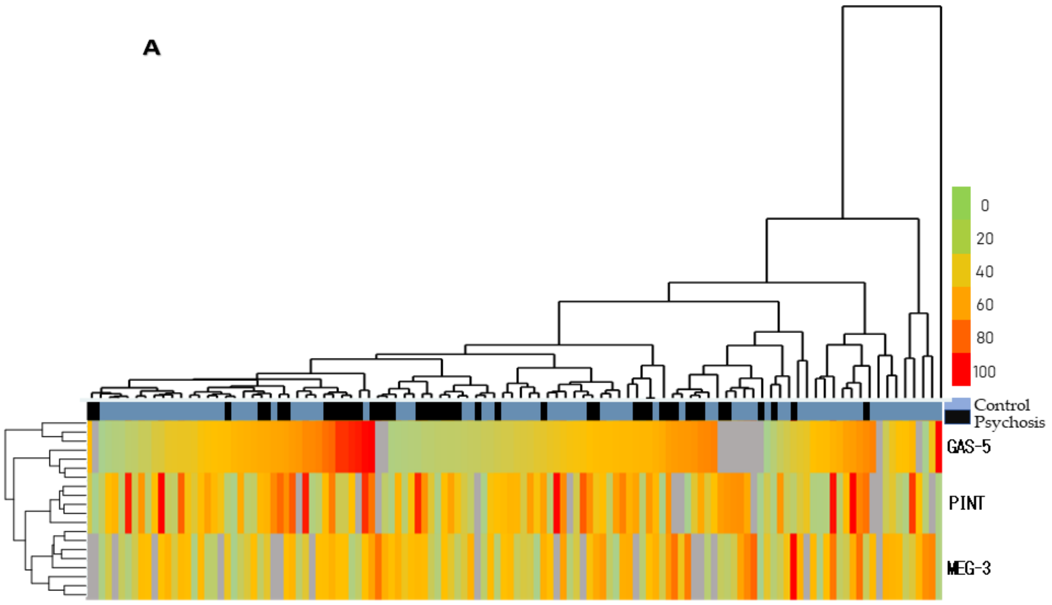

B

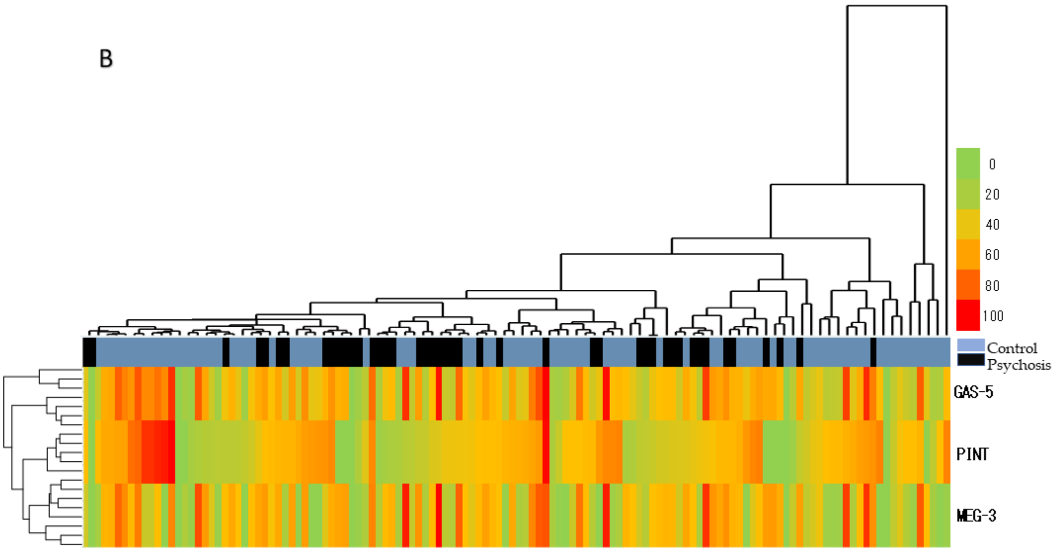

Figure S3: Dendrograms and heat map of hierarchical clustering analysis (complete-linkage) by the expression of lncRNAs MEG-3, PINT and GAS-5, (**A**) predicting clinical diagnosis and, (**B**) predicting symptom acuity

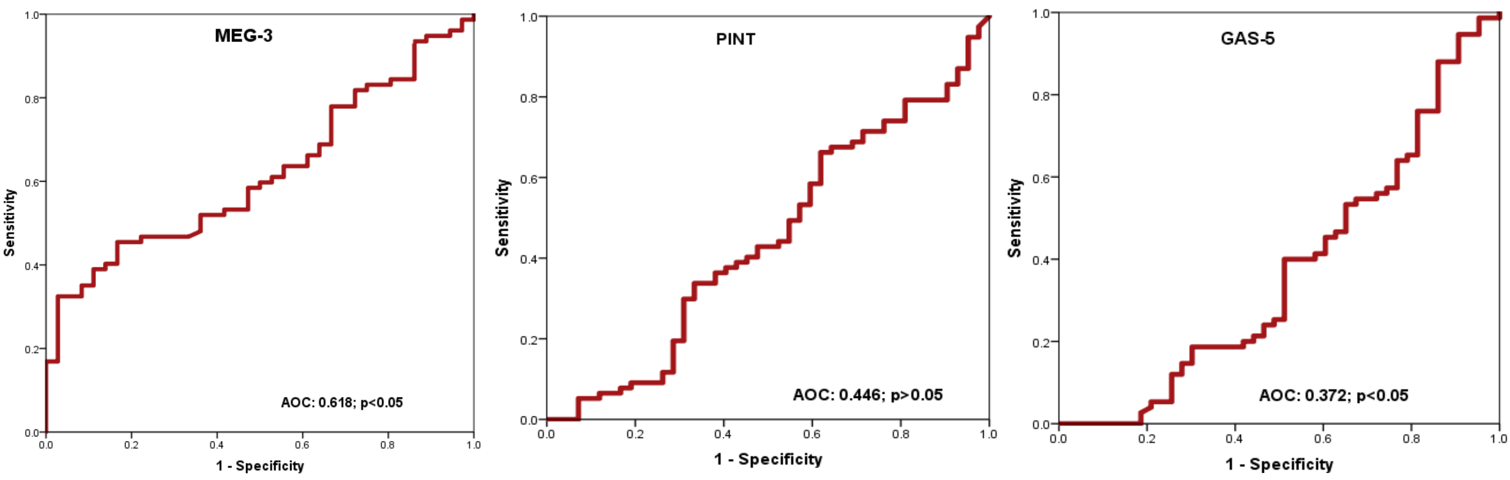

Figure S4: Receiver Operating Characteristic Curve (ROC) for predicting clinical diagnosis with lncRNAs (MEG3, PINT and GAS5) expression.
